# Tuning the Proportional–Integral–Derivative Control Parameters of Unmanned Aerial Vehicles Using Artificial Neural Networks for Point-to-Point Trajectory Approach

**DOI:** 10.3390/s24092752

**Published:** 2024-04-26

**Authors:** Burak Ulu, Sertaç Savaş, Ömer Faruk Ergin, Banu Ulu, Ahmet Kırnap, Mehmet Safa Bingöl, Şahin Yıldırım

**Affiliations:** 1 Department of Mechatronics Engineering, Erciyes University, 38039 Kayseri, Turkey; burakulu@erciyes.edu.tr (B.U.); sertacsavas@erciyes.edu.tr (S.S.); msbingol@ohu.edu.tr (M.S.B.); sahiny@erciyes.edu.tr (Ş.Y.); 2 Department of Mechanical Engineering, Erciyes University, 38039 Kayseri, Turkey; omer@erciyes.edu.tr; 3 Department of Software Engineering, Kayseri University, 38030 Kayseri, Turkey; banuulu@kayseri.edu.tr; 4 Department of Mechatronics Engineering, Nigde Omer Halisdemir University, 51240 Nigde, Turkey

**Keywords:** neural networks, trajectory control, unmanned aerial vehicles, autonomous navigation, agricultural technologies

## Abstract

Nowadays, trajectory control is a significant issue for unmanned micro aerial vehicles (MAVs) due to large disturbances such as wind and storms. Trajectory control is typically implemented using a proportional–integral–derivative (PID) controller. In order to achieve high accuracy in trajectory tracking, it is essential to set the PID gain parameters to optimum values. For this reason, separate gain values are set for roll, pitch and yaw movements before autonomous flight in quadrotor systems. Traditionally, this adjustment is performed manually or automatically in autotune mode. Given the constraints of narrow orchard corridors, the use of manual or autotune mode is neither practical nor effective, as the quadrotor system has to fly in narrow apple orchard corridors covered with hail nets. These reasons require the development of an innovative solution specific to quadrotor vehicles designed for constrained areas such as apple orchards. This paper recognizes the need for effective trajectory control in quadrotors and proposes a novel neural network-based approach to tuning the optimal PID control parameters. This new approach not only improves trajectory control efficiency but also addresses the unique challenges posed by environments with constrained locational characteristics. Flight simulations using the proposed neural network models have demonstrated successful trajectory tracking performance and highlighted the superiority of the feed-forward back propagation network (FFBPN), especially in latitude tracking within 7.52745 × 10^−5^ RMSE trajectory error. Simulation results support the high performance of the proposed approach for the development of automatic flight capabilities in challenging environments.

## 1. Introduction

In recent years, autonomous unmanned aerial vehicles (UAVs) have been given significant attention due to their enormous potential applications in civil and military fields. UAVs appear as micro-aerial robots that support a sustainable environment, offer a contactless delivery option, or can be used for hobby purposes [1,2,3,4,5]. In addition, aerial robots can be used autonomously in many applications with cameras and equipment mounted on them, such as increasing agriculture productivity, determining product maturity, detecting diseases and agricultural spraying [6]. Autonomous aerial robots should use a robust controller to perform assigned tasks with higher accuracy on specified trajectories. Many different methods are applied to control autonomous aerial robots. Masse et al. used the linear quadratic regulator (LQR) method and structured an H∞ synthesis method for aerial robot control. The results show that the H∞ method performs better than the LQR method in windy conditions. However, it presented an overview based on mathematical models and the section on actual working conditions is not mentioned [7]. Perozzi et al. utilized sliding mode control for trajectory tracking of a quadrotor. Results of numerical experiments confirmed the sliding mode control’s success in stabilizing the quadrotor under varying wind. Their study aimed to develop a control structure based on predicted wind; however, the variable conditions in the real environment were not taken into account [8]. Celen and Oniz used fuzzy logic and neural network controllers for trajectory tracking for a quadcopter. The results show that artificial neural networks (ANNs) provide stability and robustness to the drone system [9]. In addition to the mentioned control methods, control models such as model predictive control, backstepping control and model reference adaptive control are also used [10,11]. Another popular method used for drone control is the proportional–integral–derivative (PID) controller [12]. Tuning the gain parameters of the PID controller is important, although the most commonly used method for tuning these parameters is trial and error. This method is time consuming as Lee and Peng pointed out in their study. Therefore, different methods are needed for tuning parameters [13].

The importance of PID control lies in its simplicity, efficiency, and above all, how easy it is to implement. There are many PID control studies in the literature developed for trajectory control in UAVs [14]. In these studies, many of the solutions given in terms of PID control are created temporarily, meaning that priority is given to solving the task rather than providing an analysis that will reveal the limitations and advantages of the control strategy. Independent of these studies, the number of ANN-based approaches that have increased their effectiveness has been increasing rapidly, especially in recent years.

Wang et al. used RBF (radial basis function) neural network to tune the autonomous flight PID controller parameters of UAVs in adapting to different environments [15]. Gao et al. used NN-PID to control the attitude and position of a quadrotor. Simulation results show that their NN-PID algorithm is more robust than the PID algorithm [16]. In many studies, quadrotors exhibit complex nonlinear dynamics resulting from complex interactions between multiple rotors and their effects on both translational and rotational motion. PID controllers designed by classical methods for linear systems have difficulty in accurately modeling and handling the nonlinear behavior of quadrotors. Therefore, superior methods such as artificial intelligence are needed for tuning PID parameters.

In this study, an ANN-based PID control parameter adjustment method is proposed for the autonomous flying robot system developed to determine the yield in an apple orchard where autotune mode is not possible [17]. This system is controlled with three different PID control structures for roll, pitch and yaw movements. Thus, the adaptation of the quadrotor to linear PID parameters is facilitated by using nonlinear behavioral parameters in ANN training. For this purpose, the flying robot system, which has a script coded in Python, performed flight simulations with 200 different randomly determined PID parameter combinations in the Mission Planner simulation environment to which it is connected via SITL, and a data set with position data as input and PID parameters as output is created. Simulations performed on the trajectory used as a reference while creating the data set show that changing the PID parameters of the yaw control does not cause a significant change in the trajectory error. Therefore, a neural network predictor model for yaw control is not developed in this study. The data set obtained for roll and pitch controllers is used for models developed with three different ANN algorithms. As a result, test flights are carried out in the Mission Planner simulation environment of the quadrotor system with the PID gain parameters estimated by three different ANN models, and the performance and error values of the estimated PID parameters were observed. Simulation results are presented comparatively in tables and graphics.

## 2. Methodology

**Figure 1:**
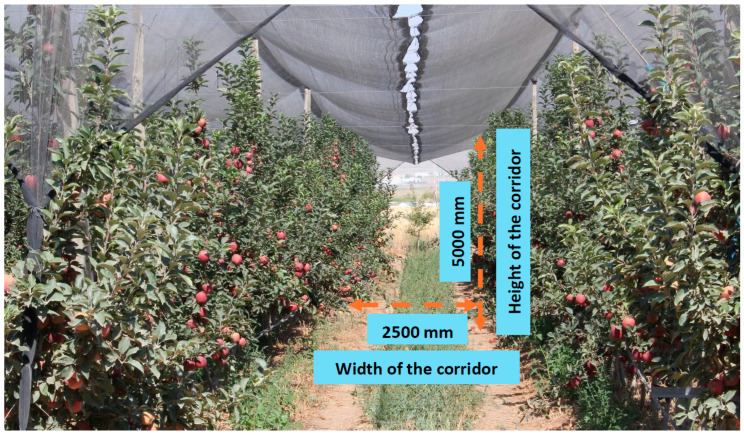
Apple orchard where flying robotic system is used, Yeşilhisar, Kayseri, Turkey.

Using the conventional approach of autotune mode to establish control parameters for the quadrotor robotic system is deemed unsuitable, particularly in an apple orchard’s narrow and sparsely covered corridors, as illustrated in Figure 1. Autotune mode is frequently used in studies to determine PID control parameters. Mini aerial vehicles using this mode automatically determine the optimum K_p_, K_i_ and K_d_ gain parameters in large and open areas, with movements similar to the dance of bees in the air. However, it is not possible to apply the method in the apple orchard where the study will be carried out to determine the yield since the apple orchards have narrow corridors and the upper parts are covered with thin cover. For this reason, in this study, the K_p_, K_d_ and K_i_ gain parameters are determined with artificial intelligence methods in the determined trajectories and the system performance is evaluated. In the study, the determination of K_p_, K_d_ and K_i_ gain parameters is carried out in a simulation environment, and the trajectory tracking performance of the quadrotor system is examined with the obtained gain parameters.

**Figure 2:**
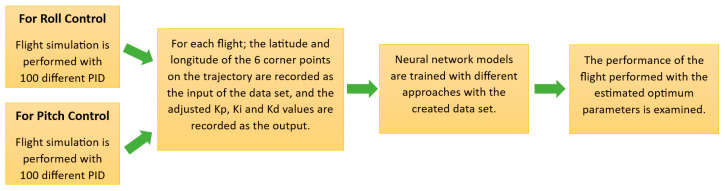
Flow chart description of the proposed methodology.

This section describes the developed system with a broad explanation. Firstly, software specifications are outlined in detail. The modified standard PID controller is described with tuned gain parameters. The general methodology of this article is given in Figure 2. The study, seen in Figure 2, consists of four basic stages.

### 2.1. Software Specifications

Ardupilot software (Copter-4.4.0) is embedded in the controller to run flight commands and manage the flight. Through this software, precise control of the quadrotor can be achieved with PID control parameters determined through the flight planning program. The high accuracy of the simulation software is demonstrated by comparing simulated and experimental trajectories for three different UAVs [18]. In this study, Mission Planner is used as the flight planning and simulation program. Furthermore, the quadrotor dynamics [19] required for flight are provided by embedded libraries in the Mission Planner open source software [20].

**Figure 3:**
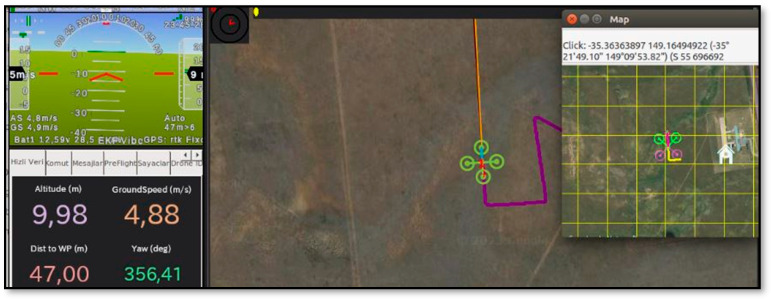
View of one of the simulation flights on the map.

In addition to referenced studies, flight trajectory is created for simulation to compare trajectory tracking performances with different K_p_, K_i_ and K_d_ parameters. For the quadrotor to track this trajectory, which is similar to the corridors in the apple orchard, a mission program is coded in Python using the Dronekit library. Software in the Loop (SITL) is used to simulate systems operating in real time [21]. Using this program, each simulation flight performed with different parameters is tracked on the map as shown in Figure 3, and location data is recorded. These data are then processed and a data set is created for training the neural network model.

### 2.2. Standard PID Controller System

PID controllers have been used for a wide variety of systems. Today, PID control is also used in many drone studies [22]. The continuous time PID controller is defined by Equation (1) [23]. To drive a plant output y(t), towards a reference signal r(t), the control input u(t), is calculated by a closed loop PID controller, which uses the error in the output, that is, *e*(*t*) = r(t) − y(t) [24]. In Equation (1), *K_p_* is the proportional gain, *K_i_* is the integral gain and *K_d_* is the derivative gain.

(1) $$u\left(t\right)=K_{p}\cdot e\left(t\right)+K_{i}\cdot \int e\left(t\right)dt+K_{d}\cdot \frac{d}{dt}e(t)$$

Appropriate coefficient values must be adjusted for the PID controller parameter to operate correctly. Although the Ziegler Nichols method [25] is the most widely used method for tuning these parameters in many systems, it is not easy to apply in such unique systems. Due to the high degree of non-linearity in these systems, it is difficult to modify the PID controller’s gain parameters when there are external disturbances or the system parameters are changing. As a result, the PID controller’s performance is reduced [26].

**Figure 4:**
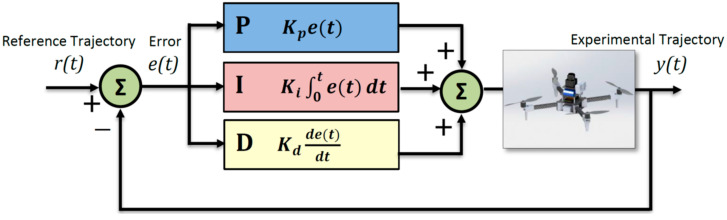
Standard PID control system.

MAVs perform their flights according to specific modes. This study uses Auto and Guided modes for the quadrotor to perform the task fully autonomously. Auto mode is used when the flight program is uploaded to the controller, and Guided mode is used when it is managed externally through the program written with Dronekit. In both modes, position errors must be minimized by continuous monitoring of a controller so the quadrotor system can follow the determined trajectory. This system is controlled by three different PID control structures for roll, pitch and yaw movements. The PID control model of UAVs is shown schematically in Figure 4 for UAV movements.

*K_p_*, *K_i_* and *K_d_* parameters must be tuned for each control model before the flight. This tuning can be performed by the trial-and-error method based on experience or adjusted autonomously in autotune mode, a mode developed for this type of MAV. If there is a suitable and sufficient area for flight, automatically tuning the PID controller gain parameters gives a more effective result. In this mode, the UAV moves autonomously in a wide-area trajectory, similar to the dance of bees, according to a determined algorithm. During this movement, K_p_, K_i_ and K_d_ parameters are tuned in the most optimal way.

However, it is not possible to use the autotune mode because the micro-UAV designed in this study will perform its movement in a narrow and covered environment among trees. For this reason, an artificial neural network (ANN) model has been developed to determine the K_p_, K_i_ and K_d_ parameters.

In the simulation environment, a trajectory was determined by reference to the row size of the tree array where the experimental study will be carried out. This trajectory is autonomously followed with datasets consisting of 100 different K_p_, K_i_ and K_d_ parameters determined separately for the roll and pitch control models. During the sample preparation, it is experienced that 100 samples in the dataset are sufficient to extract the characteristics between the position-PID parameters. Changing the PID parameters of the yaw control does not cause a significant change in the trajectory error. Therefore, there is no need for an optimization for the yaw control model in the simulation flights, and the default values are used in all flights. The default values for the gain parameters are 0.2 for K_p_, 0.02 for K_i_ and 0.002 for K_d_.

In this method, 200 flight simulations are performed according to the parameter changes in two different control models. The K_p_, K_i_ and K_d_ parameters adjusted in each simulation are used as output values for the samples in the training of the ANN model. These values are determined randomly through the program and the K_p_, K_i_ and K_d_ parameters of one of the roll and pitch control models were changed while the others were kept constant. In this way, it is aimed to determine the optimum K_p_, K_i_ and K_d_ control parameters for each movement type.

### 2.3. Proposed Artificial Neural Network (ANN)

For tuning PID parameters, several offline methods are available. In this section, the critical ideas of ANN, supervised learning and system identification methodologies are introduced and analyzed in relation to the theoretical development and applications of optimal PID controllers [27]. ANNs can offer flexible solutions to non-linear problems with their structure similar to the human brain.

ANNs where data flow only in the forward direction are feed-forward networks. ANNs with connections that allow data to flow both forward and backward are called feedback neural networks. One of the network structures used in this study is the feed-forward back propagation network (FFBPN). The FFBPN is a method of supervised learning. Radial basis neural network (RBNN) and cascade forward back propagation network (CFBPN), other network structures used in the study, are also supervised learning methods.

**Figure 5:**
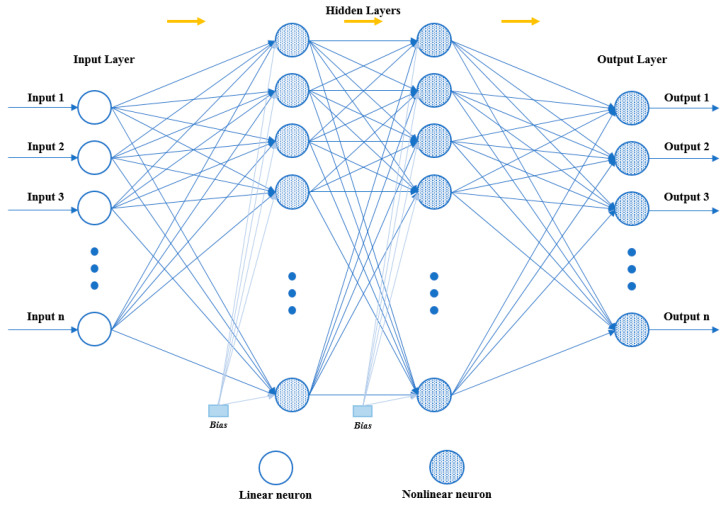
The schematic representation of the FFBPN model with signal flow direction.

The schematic representation of the ANN model trained to estimate the K_p_, K_i_ and Kd parameters for the FFBPN model is given in Figure 5.

The weights between input and hidden layers are updated as Equation (2) in FFBPN, where η is the learning rate and α is the momentum term. *E_2_*(*t*) is the propagation error between hidden and input layers. *E_1_*(*t*) is the error between experimental and neural network output signals [28,29].

(2) $$∆\;W_{ij}\left(t\right)=-\eta \frac{\partial E_{2}\left(t\right)}{\partial W_{ij}}+\alpha ∆W_{ij}(t-1)$$

The weights between the hidden and output layers are updated as Equation (3).

(3) $$∆\;W_{jn}\left(t\right)=-\eta \frac{\partial E_{1}\left(t\right)}{\partial W_{jn}}+\alpha ∆W_{jn}(t-1)$$

**Figure 6:**
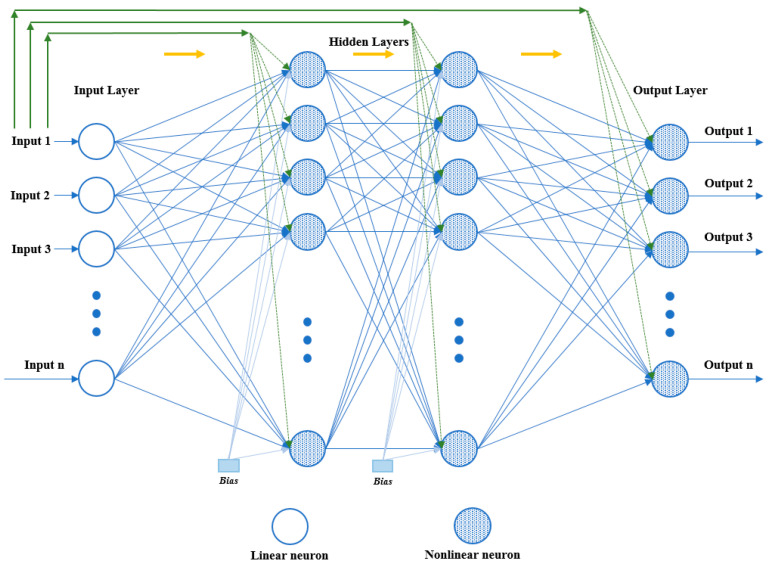
The schematic representation of the CFBPN model.

The CFBPN model is similar to a FFBPN model in using the backpropagation algorithm for weight updating. However, a fundamental characteristic of this network is that every layer of neurons is associated with all preceding layers of neurons. As other feed-forward networks, CFBPN has one or more interconnected hidden layers and activation functions. Each neuron has a bias of its own and each connection has a specific weight [30,31]. The schematic representation of the CFBPN model is given in Figure 6.

Each combination in the CFBPN learning sample (p_q_, d_q_) is calculated as follows [32]:

*p_q_* inputs are propagated forward through the layers of the m-layer neural network by Equation (4):

(4) $$a^{0}=p_{q};a^{k}=f^{k}\left(W^{k}a^{k-1}-b^{k}\right),\;k=1,\ldots ,M$$

where *p_q_* is input, *a* is cell output, *b* is bias and *w* is weight.

Back propagate the sensitivities through the layers by Equation (5):

(5) $$\delta^{M}=-2F^{\prime M}\left(n^{M}\right)\left(d_{q}-a^{M}\right);\delta^{k}=-F^{\prime k}\left(n^{k}\right)\left(W^{{k+1}^{T}}\right)\delta^{k+1},\;k=M-1,\ldots ,1$$

Modify the biases and weights by Equations (6) and (7), respectively:

(6) $${\Delta b}^{k}=\eta \delta^{k},\;k=1,\ldots ,M$$

(7) $${\Delta W}^{k}=-\eta \delta^{k}{\left(a^{k-1}\right)}^{T},\;k=1,\ldots ,M$$

These steps continue until the stopping criterion is reached.

**Figure 7:**
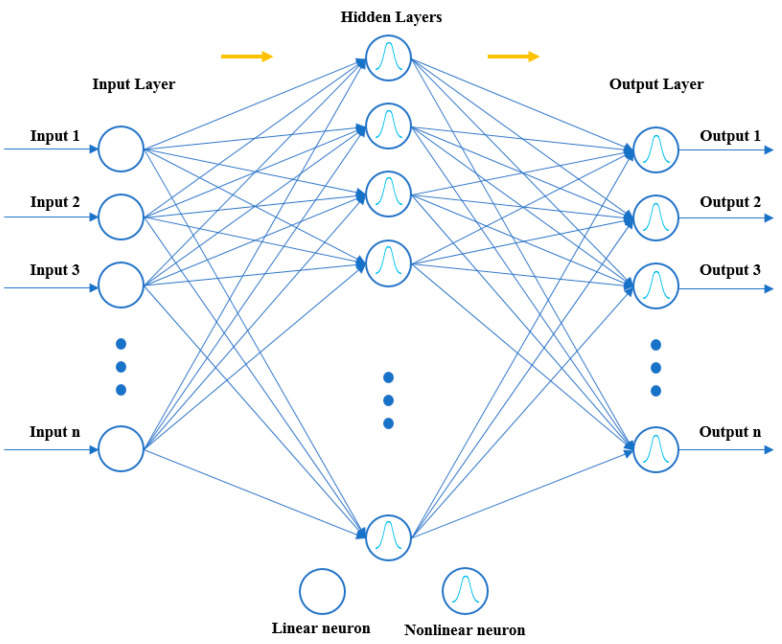
The schematic representation of the RBNN model.

The nonparametric estimation of multidimensional functions using sparse amounts of training data is a common application for RBNN. Fast and thorough training of RBNNs makes them effective [33,34]. RBNNs have many benefits, including strong global approximation ability, no local minimum difficulties and fast learning speed [35]. FFBPNs can have one or multiple hidden layers, while RBNNs have only one. The input layer, which is the first layer of the RBNN, just serves to transfer information and does not process the input data in any way. The second layer is a hidden layer. The third layer is the output layer, which will linearly transform the input data and then the output [36]. The schematic representation of the RBNN model is given in Figure 7.

The objective function in RBNN can be defined by Equation (8), where h_i_ is the height value of sample *i* [37].

(8) $$E=\frac{1}{2}\sum_{i=1}^{n}{\left(h_{i}-f(x_{i}\right)}^{2})$$

The *f*(*x*) given in Equation (8) can be defined as in Equation (9).

(9) $$f(x)=\sum_{i=1}^{n}\omega_{i}\phi (∥x-c_{i}∥)$$

where ***n*** is the number of neurons in the hidden layer, $\omega_{i}$ ∈ W is the weight of neuron i in the linear output neuron, *c_i_* denotes the center vector of the neuron i, ϕ (.) denotes the nonlinear function that is a multiquadratic function in Equation (10).

(10) $$\phi (r)=r\sqrt{1+\xi^{2}}$$

where *r* denotes the distance between unknown and known data, and *ξ* is a smoothing factor between 0 and 1. As a result, the formula used to calculate the weights in the network structure is given in Equation (11).

(11) $$W\left(t+1\right)=W\left(t\right)-\eta \cdot \frac{\partial E}{\partial W}=W\left(t\right)+\eta \cdot \sum_{i=1}^{n}\left(h_{i}-f\left(x_{i}\right))\phi (∥x-c_{i}∥\right)$$

The latitude and longitude values (12 pieces) of the corner points determined from the position data recorded at 2 s intervals during the flight are used as input in the training of the ANN model. The output of the ANN model is the parameters K_p_, K_i_ and K_d_. The performance of the ANN models for the roll and pitch control models are examined in detail in the Results section.

## 3. Simulation Results

### 3.1. Estimation of PID Parameters

In the study, three different artificial neural network models, namely feed-forward back propagation neural network, cascade-forward back propagation neural network and radial basis neural network, are designed to determine the PID control parameters of the flying robot system. The designed models are trained with the trajectory points-PID gain values data set obtained in the Mission Planner simulation environment. Z-score normalization is applied to the entire data set for the training process. PID parameters for the reference trajectory are estimated with the trained artificial neural network models. The data set allocated for 100 rolling and 100 pitching values is divided into three groups as 70% train, 15% test and 15% validation for FFBPN and CFBPNN. This separation is performed randomly among the data. Then, flight tests are carried out in the same simulation environment with the obtained parameters and flight performances are compared in terms of latitude, longitude and altitude.

**Figure 8:**
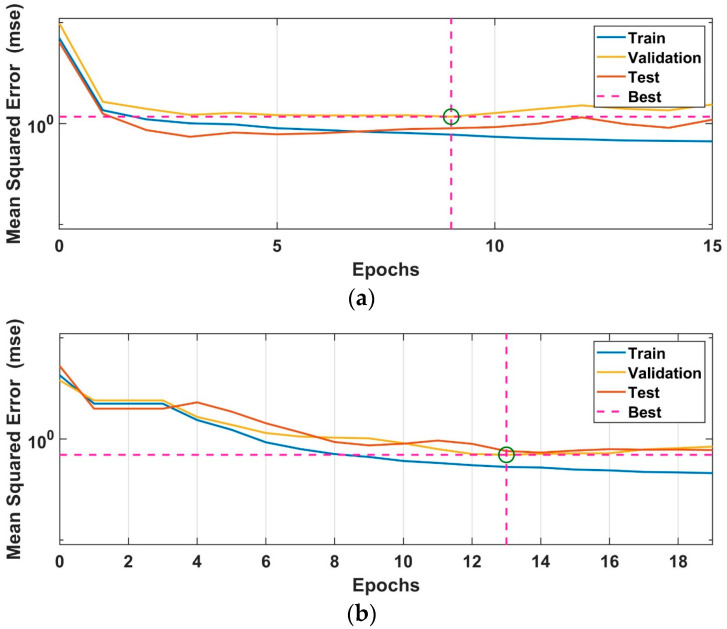
Training performance graphs of FFBPN models giving the best training results used to estimate PID parameters of the controllers for (**a**) rolling and (**b**) pitching.

FFBPN and CFBPN artificial neural network models are designed as twelve inputs, three outputs and two hidden layers with eight neurons each. In the training process of both models, three training algorithms, the Levenberg–Marquart (trainlm), scaled conjugate gradient (trainscg) and BFGS quasi-newton (trainbfg), and three activation functions (logsig, radbas and tansig) are used. The training algorithm and activation function that gave the lowest training error are determined by the grid search method to be used in performance tests. Training parameters of artificial neural network models are given in Table 1. MSE values of FFBPN models and estimated PID parameters for the rolling and pitching controllers are given in Table 2. Figure 8 shows the performance plots of FFBPN neural network models with the best training MSE values for rolling and pitching control, respectively.

**Figure 9:**
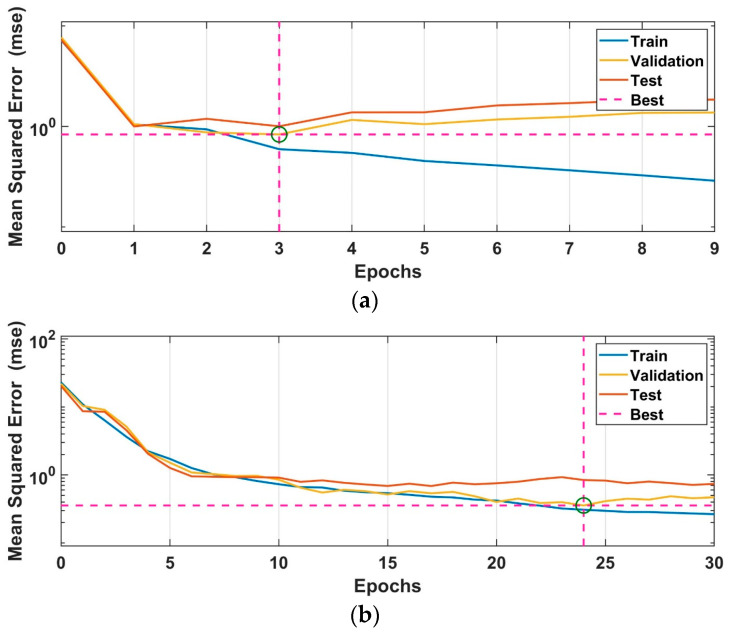
Training performance graphs of CFBPN models giving the best training results used to estimate PID parameters of the controllers for (**a**) roll control and (**b**) pitch control.

MSE values of CFBPN models and estimated PID parameters for the rolling and pitching controllers are given in Table 3. Figure 9 shows the performance plots of CFBPN neural network models with the best training MSE values for rolling and pitching control.

**Figure 10:**
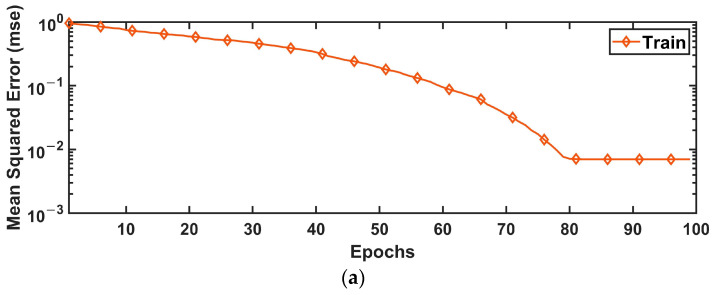

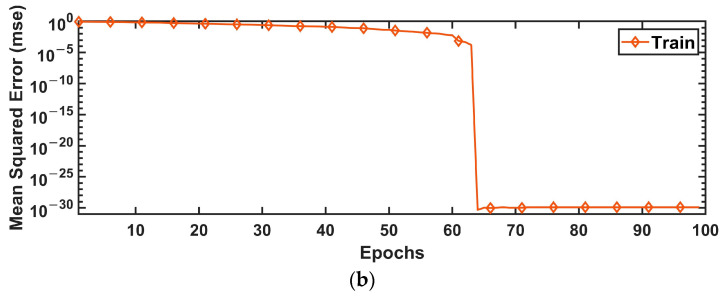
Training performance graphs of RBNN model used to estimate PID parameters of the controllers for (**a**) rolling and (**b**) pitching.

Finally, the RBNN model is designed and training procedures are carried out to determine the control parameters of both control approaches. Although this artificial neural network model also has twelve inputs and three outputs, it has 100 neurons in its single hidden layer. The spread value of the model is selected as 0.1. Training MSE values and control gain values estimated with the trained network are given in Table 4. Additionally, the training performance graphs of RBNN network trained for rolling and pitching control are shown in Figure 10.

### 3.2. Flight Performance Tests

A reference flight trajectory is designed in the Mission Planner program to analyze the performance of PID control gain parameters obtained with artificial neural network models in roll and pitch control. Flights are carried out with each PID value and the tracking performance of the reference latitude, longitude and altitude values are analyzed with four error metrics. The mathematical expressions of the error metrics RMSE (root mean square error), MSE (mean square error), MAE (mean absolute error) and MAPE (mean absolute percentage error) used for this purpose are given in Equations (12)–(15). RMSE (root mean square error) is the square root of the mean of the squares of the differences between the actual and reference values, giving greater weight to larger errors. MSE (mean squared error) is the average of the squares of the differences between the actual and reference values, providing an average value according to the error squares. MAE weights all errors equally by averaging the absolute values of the error amounts, thus reducing the weight of larger errors. MAPE evaluates the relative accuracy of actual values relative to the reference by providing a percentage measure of errors, making it easier to compare data at different scales.

(12) $$RMSE=\sqrt{\frac{1}{n}\sum_{i=1}^{n}{\left(y_{i}-r_{i}\right)}^{2}}$$

(13) $$MSE=\frac{1}{n}\sum_{i=1}^{n}{\left(y_{i}-r_{i}\right)}^{2}$$

(14) $$MAE=\frac{1}{n}\sum_{i=1}^{n}\left|y_{i}-r_{i}\right|$$

(15) $$MAPE=\frac{1}{n}\sum_{i=1}^{n}\left|\frac{y_{i}-r_{i}}{y_{i}}\right|\cdot 100$$

**Figure 11:**
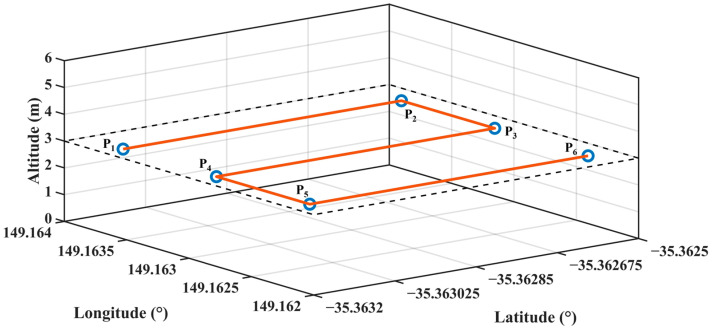
Reference trajectory and corner points (P_1_: Start point, P_2_–P_5_: Trajectory points and P_6_: End point) to be used for performance testing.

In the equations, *n* is the number of samples, *y_i_* is the ith actual output value, and *r_i_* is the ith reference value. The reference trajectory points determined for the performance test are given in Table 5 and the reference trajectory is given in Figure 11. Additionally, rolling and pitching PID controller gain values are given in Table 6 for all artificial neural network models.

**Figure 12:**
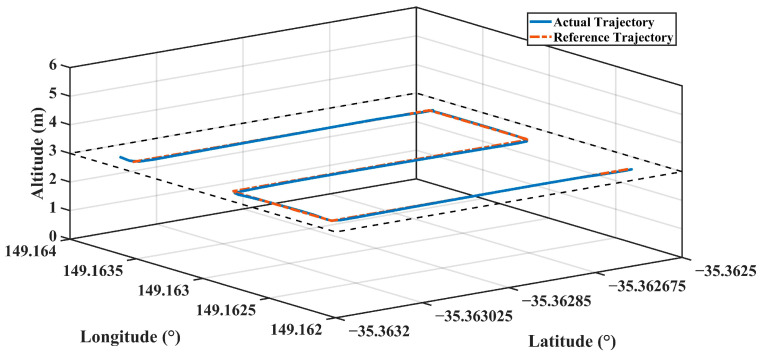
The actual and reference trajectory variations of the flight performed with PID parameters estimated with FFBPN.

**Figure 13:**
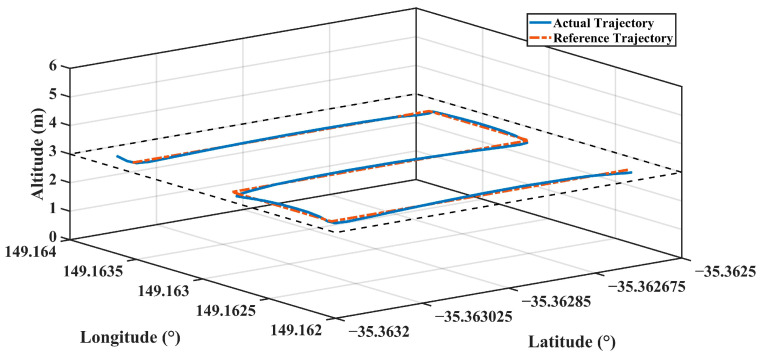
The actual and reference trajectory variations of the flight performed with PID parameters estimated with CFBPN.

**Figure 14:**
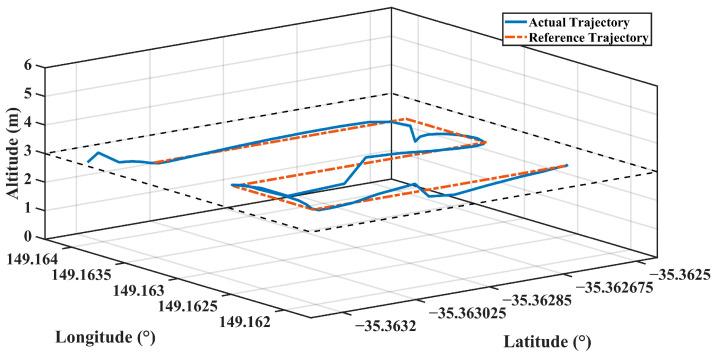
The actual and reference trajectory variations of the flight performed with PID parameters estimated with RBNN.

The actual and reference trajectories of the flight realized with PID parameters estimated with FFBPN are given in Figure 12, CFBPN in Figure 13, RBNN in Figure 14. In addition, error metrics for latitude, longitude and altitude values, which are position information for each flight, are given in Table 7.

**Figure 15:**
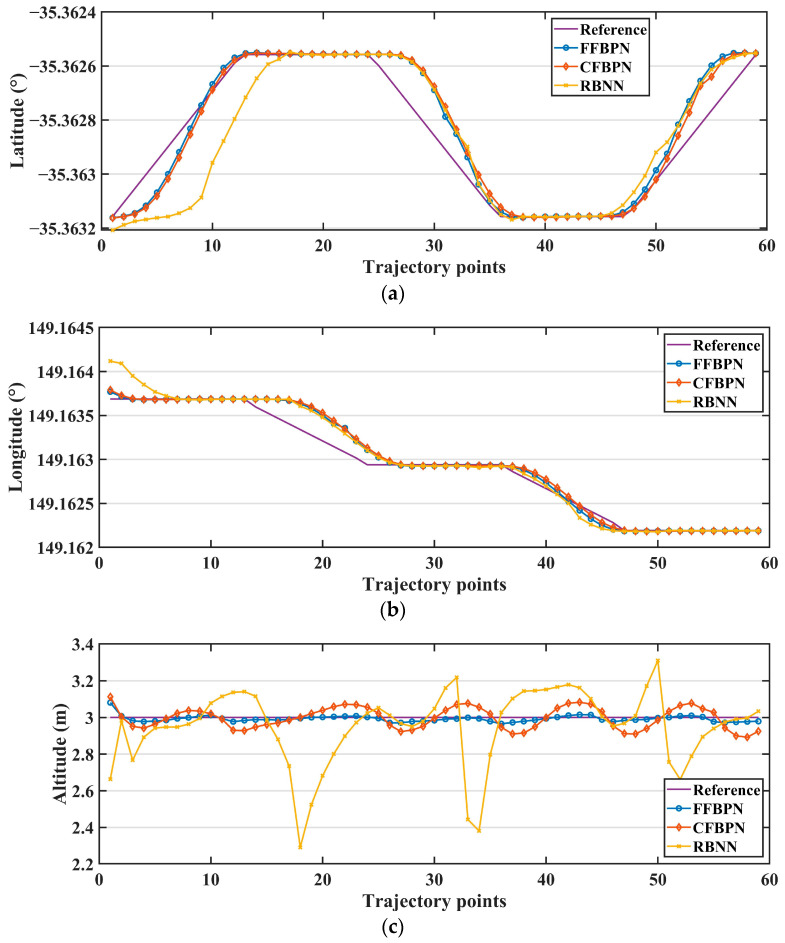
The reference and actual (**a**) latitude, (**b**) longitude and (**c**) altitude values.

**Figure 16:**
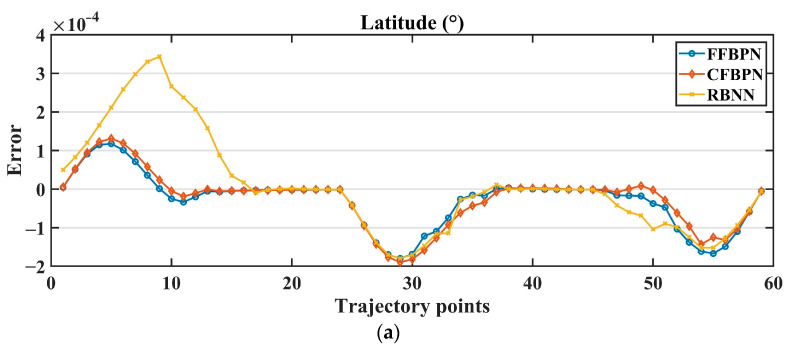

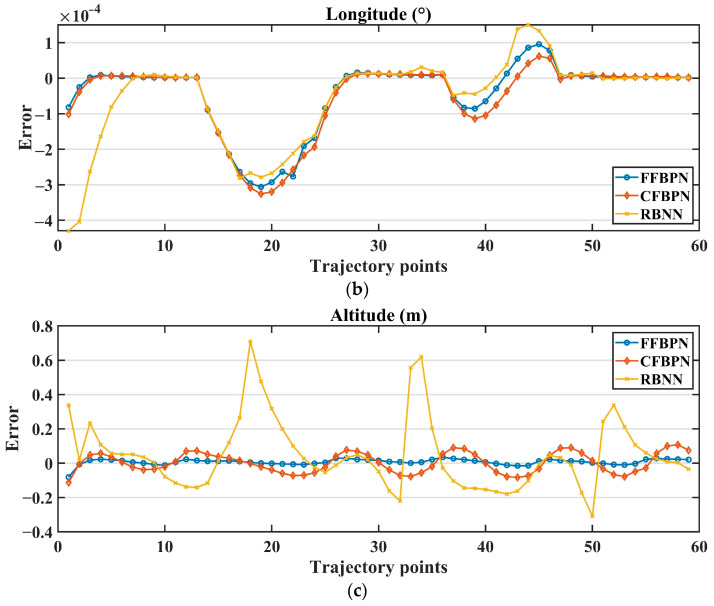
The RMSE error graphs of (**a**) latitude, (**b**) longitude and (**c**) altitude values.

The reference and actual latitude, longitude and altitude values for the flights performed with all control parameters obtained with the three ANN models are shown in Figure 15, and the RMSE error graphs of these coordinate values are shown in Figure 16.

When the error metrics and graphs are examined, the results are quite close in flights where PID parameters obtained with FFBPN and CFBPN models are used. However, the performance of the parameters obtained with RBNN appears to be unsuccessful, especially in terms of latitude and altitude values. Especially, fluctuations in the flying robot system’s altitude values cannot be ignored. Although the training error of the RBNN model is lower than the other two models, the fact that it is not successful in the test results reveals the possibility of overfitting. Therefore, it is understood that it cannot produce a robust response to external situations. This shows that the RBNN model is unsuitable for this specific task compared to the other two.

When FFBPN and CFBPN are compared with each other, it is seen that FFBPN parameters provide more successful tracking. Again, although the train errors of these models are quite close to each other, the test results reveal that the train performance should be evaluated in terms of its own hyperparameters within each network model. When the flight performance of the parameters estimated by the two models is examined, the models do not reveal very different results in terms of latitude and longitude values. In contrast, in the flight study conducted with CFBPN, it is observed that there is an increase in altitude on long straight roads and a decrease in corner points. This shows that the FFBPN model is more successful than others in predicting altitude changes.

Additionally, feed-forward networks include only forward connections between layers, whereas cascade-forward networks also have direct connections of the inputs and outputs of each layer to subsequent layers. While this enables cascade-forward networks to model more complex relationships by capturing longer-range dependencies in the data set, it may cause undesirable lengths of training time and the risk of overfitting in non-complex data sets. Therefore, it is necessary to consider each artificial neural network model by assessing the nature and properties of the data set.

## 4. Discussion and Conclusions

In this study, the optimum PID gain parameters of the micro air vehicle planned to fly in narrow apple orchard corridors are estimated with ANN. For this purpose, a data set is created from latitude, longitude and altitude data of flights performed with PID parameters randomly determined between significant lower and upper values. Since the realization of flights with random PID parameters in the real environment involves high risks, the flights are carried out in a simulation environment and artificial neural network models that make predictions based on the position data followed by the micro aerial vehicle according to parameter changes are developed with the obtained data set. Three different neural network models are designed with FFBPN, CFBPN and RBNN architectures, which are frequently used to determine the optimum PID gain parameters. The neural network models are trained with different parameters and their performances are analyzed. The MSE values obtained show significant differences between the models. In addition, in order to test the success of the developed neural network models, simulation flights are performed using the PID gain parameters obtained with the models showing the best training performance. In the flight simulations, both FFBPN and CFBPN models exhibited successful trajectory tracking performance in relation to their training performance, while FFBPN exhibited superior altitude tracking capabilities. As a result, it has been demonstrated that the PID parameters of a micro aerial vehicle can be tuned with artificial neural networks instead of randomizing them and an alternative solution has been proposed for challenging conditions where autotune mode cannot be used.

## Figures and Tables

**Table 1:** FFBPN and CFBPN training parameters.

Performance Function	MSE
Maximum Number of Iterations (Epochs)	1000
Minimum Gradient	10^−6^
Damping Factor (mu) (trainlm)	0.15

**Table 2:** MSE values of FFBPN models and estimated PID parameters for the rolling and pitching controllers.

Control Type	Training Algorithm	Activation Function	MSE	K_p_	K_i_	K_d_
Rolling Control	Levenberg–Marquardt	tansig	0.9077	2.1101	2.0188	0.5137
		radbas	0.8778	2.8903	1.9165	0.7645
		logsig	0.9184	2.0596	2.0874	0.5805
	Scaled Conjugate Gradient	tansig	0.9051	2.2497	1.3240	0.4064
		radbas	1.3643	1.9666	2.3850	0.2320
		logsig	0.9533	2.0811	1.8916	0.5350
	BFGS Quasi-Newton	tansig	0.9735	2.1330	2.0543	0.4461
		radbas	0.8526	2.0328	2.2562	0.7115
		logsig	0.9740	2.4656	2.3052	0.5739
Pitching Control	Levenberg–Marquardt	tansig	0.6101	2.4077	1.8095	0.6013
		radbas	0.7179	3.5663	2.0397	1.3567
		logsig	0.6539	1.9875	2.6991	0.8251
	Scaled Conjugate Gradient	tansig	0.9665	1.7854	2.2571	0.4842
		radbas	0.5881	2.7480	2.2292	0.8836
		logsig	0.7993	2.8513	2.4859	0.4041
	BFGS Quasi-Newton	tansig	0.7017	2.4867	2.4330	1.1562
		radbas	0.6019	1.4790	2.5836	0.5073
		logsig	0.9760	2.3690	2.2313	0.7147

**Table 3:** MSE values of CFBPN models and estimated PID parameters for the rolling and pitching controllers.

Control Type	Training Algorithm	Activation Function	MSE	K_p_	K_i_	K_d_
Roll Control	Levenberg–Marquardt	tansig	0.6911	1.7155	0.6107	1.1281
		radbas	0.9593	2.8376	1.3909	0.8655
		logsig	0.8521	2.5103	1.0876	0.6433
	Scaled Conjugate Gradient	tansig	0.9067	2.6215	1.5810	0.6680
		radbas	0.8592	2.4244	2.3351	0.6117
		logsig	0.9466	1.9622	2.2506	0.4117
	BFGS Quasi-Newton	tansig	0.8748	2.3739	1.5228	1.2769
		radbas	0.9827	1.6846	1.3402	0.4403
		logsig	0.9478	2.4340	1.1644	0.5635
Pitch Control	Levenberg–Marquardt	tansig	0.6539	2.5264	1.9099	1.7538
		radbas	0.4553	2.0911	2.5904	1.1806
		logsig	0.6549	1.4632	1.3870	1.1121
	Scaled Conjugate Gradient	tansig	0.5553	1.5742	2.1896	0.7747
		radbas	0.7048	2.3274	3.4386	1.7152
		logsig	0.6983	2.4532	1.6562	2.3723
	BFGS Quasi-Newton	tansig	0.3947	1.2427	0.6283	0.7927
		radbas	0.6913	2.5531	1.9590	1.2485
		logsig	0.7019	1.9238	1.2242	0.0080

**Table 4:** MSE values of RBNN model and estimated PID parameters for the roll and pitch controllers.

Control Type	MSE	K_p_	K_i_	K_d_
Rolling Control	0.0070	2.8264	3.2051	0.8674
Pitching Control	1.2665 × 10^−30^	0.7937	1.7214	0.0803

**Table 5:** Latitude, longitude and altitude values of the reference trajectory points to be used for performance testing.

	P_1_	P_2_	P_3_	P_4_	P_5_	P_6_
**Latitude (°)**	−35.363157	−35.362557	−35.362557	−35.363157	−35.363157	−35.362557
**Longitude (°)**	149.163687	149.163687	149.162939	149.162938	149.162191	149.162190
**Altitude (m)**	3	3	3	3	3	3

**Table 6:** Roll and pitch PID controller gain values for all artificial neural network models.

	Roll Control			Pitch Control		
	K_p_	K_i_	K_d_	K_p_	K_i_	K_d_
FFBPN	2.0328	2.2562	0.7115	2.7480	2.2292	0.8836
CFBPN	1.7155	0.6107	1.1281	1.2427	0.6283	0.7927
RBNN	2.8264	3.2051	0.8674	0.7937	1.7214	0.0803

**Table 7:** Error metrics for latitude, longitude and altitude values.

NN Model	Coordinate	RMSE	MSE	MAE	MAPE
FFBPN	Latitude	7.52745 × 10^−5^	5.66626 × 10^−9^	4.90424 × 10^−5^	8.16141 × 10^−5^
	Longitude	0.00011	1.16874 × 10^−8^	6.04816 × 10^−5^	2.87345 × 10^−5^
	Altitude	0.01878	0.00035	0.01422	0.25593
CFBPN	Latitude	7.57585 × 10^−5^	5.73935 × 10^−9^	4.90346 × 10^−5^	6.97564 × 10^−5^
	Longitude	0.00011	1.311565 × 10^−8^	6.43695 × 10^−5^	3.49091 × 10^−5^
	Altitude	0.05800	0.003364	0.05081	0.10621
RBNN	Latitude	0.00013	1.658104 × 10^−8^	8.98963 × 10^−5^	2.22769 × 10^−5^
	Longitude	0.00014	1.823412 × 10^−8^	8.10415 × 10^−5^	3.63931 × 10^−5^
	Altitude	0.20918	0.043756	0.14407	1.62034

## Data Availability

The data presented in this study are available on request from the corresponding author.

## References

[1] Almeida D.R.A., Broadbent E.N., Zambrano A.M.A., Wilkinson B.E., Ferreira M.E., Chazdon R., Meli P., Gorgens E.B., Silva C.A., Stark S.C. (2019). Monitoring the structure of forest restoration plantations with a drone-lidar system. Int. J. Appl. Earth Obs. Geoinf..

[2] Mohsan S.A.H., Khan M.A., Noor F., Ullah I., Alsharif M.H. (2022). Towards the Unmanned Aerial Vehicles (UAVs): A Comprehensive Review. Drones.

[3] Kim I.-H., Jeon H., Baek S.-C., Hong W.-H., Jung H.-J. (2018). Application of Crack Identification Techniques for an Aging Concrete Bridge Inspection Using an Unmanned Aerial Vehicle. Sensors.

[4] Chiang W.-C., Li Y., Shang J., Urban T.L. (2019). Impact of drone delivery on sustainability and cost: Realizing the UAV potential through vehicle routing optimization. Appl. Energy.

[5] Foehn P., Brescianini D., Kaufmann E., Cieslewski T., Gehrig M., Muglikar M., Scaramuzza D. (2022). AlphaPilot: Autonomous drone racing. Auton. Robot..

[6] Tao H., Feng H., Xu L., Miao M., Long H., Yue J., Li Z., Yang G., Yang X., Fan L. (2020). Estimation of Crop Growth Parameters Using UAV-Based Hyperspectral Remote Sensing Data. Sensors.

[7] MassÉ C., Gougeon O., Nguyen D.-T., SaussiÉ D. Modeling and Control of a Quadcopter Flying in a Wind Field: A Comparison Between LQR and Structured ℋ_∞_ Control Techniques. Proceedings of the 2018 International Conference on Unmanned Aircraft Systems (ICUAS).

[8] Perozzi G., Efimov D., Biannic J.-M., Planckaert L. (2018). Trajectory tracking for a quadrotor under wind perturbations: Sliding mode control with state-dependent gains. J. Frankl. Inst..

[9] Celen B., Oniz Y. Trajectory Tracking of a Quadcopter Using Fuzzy Logic and Neural Network Controllers. Proceedings of the 6th International Conference on Control Engineering & Information Technology (CEIT).

[10] Wei P., Chan S.N., Lee S., Kong Z. (2019). Mitigating ground effect on mini quadcopters with model reference adaptive control. Int. J. Intell. Robot. Appl..

[11] Rothe J., Zevering J., Strohmeier M., Montenegro S. (2020). A Modified Model Reference Adaptive Controller (M-MRAC) Using an Updated MIT-Rule for the Altitude of a UAV. Electronics.

[12] Pérez I.C., Flores-Araiza D., Fortoul-Díaz J.A., Maximo R., Gonzalez-Hernandez H.G. Identification and PID control for a quadrocopter. Proceedings of the International Conference on Electronics, Communications and Computers (CONIELECOMP).

[13] Lee C.L., Peng C.C. (2021). Analytic Time Domain Specifications PID Controller Design for a Class of 2nd Order Linear Systems: A Genetic Algorithm Method. IEEE Access.

[14] Oersted H., Ma Y. (2023). Review of PID Controller Applications for UAVs. arXiv.

[15] Wang S., Li B., Geng Q. Research of RBF neural network PID control algorithm for longitudinal channel control of small UAV. Proceedings of the 10th IEEE International Conference on Control and Automation (ICCA).

[16] Gao W.N., Fan J.L., Li Y.N. (2015). Research on Neural Network PID Control Algorithm for a Quadrotor. Appl. Mech. Mater..

[17] Yıldırım Ş., Ulu B. (2023). Deep Learning Based Apples Counting for Yield Forecast Using Proposed Flying Robotic System. Sensors.

[18] Yıldırım Ş., Çabuk N., Bakırcıoğlu V. (2023). Experimentally flight performances comparison of octocopter, decacopter and dodecacopter using universal UAV. Measurement.

[19] Asadi D. (2022). Partial engine fault detection and control of a Quadrotor considering model uncertainty. Turk. J. Eng..

[20] Karachalios T., Moschos P., Orphanoudakis T. (2024). Maritime Emission Monitoring: Development and Testing of a UAV-Based Real-Time Wind Sensing Mission Planner Module. Sensors.

[21] Khaneghaei M., Asadi D., Tutsoy Ö. Software in the Loop (SIL) Simulation for an Autonomous Multirotor Flight Planning and Landing with ROS and Gazebo. Proceedings of the 7th International Symposium on Innovative Approaches in Smart Technologies (ISAS).

[22] Noordin A., Mohd Basri M.A., Mohamed Z. (2023). Real-Time Implementation of an Adaptive PID Controller for the Quadrotor MAV Embedded Flight Control System. Aerospace.

[23] Sánchez-Palma J., Ordoñez-Ávila J.L. (2022). A PID Control Algorithm with Adaptive Tuning Using Continuous Artificial Hydrocarbon Networks for a Two-Tank System. IEEE Access.

[24] Pal A.K., Nestorović T. Artificial Intelligence Neural Network Approach to Self Tuning of a Discrete-Time PID Control System. Proceedings of the 9th International Conference on Systems and Control (ICSC).

[25] Rodríguez-Abreo O., Rodríguez-Reséndiz J., Fuentes-Silva C., Hernández-Alvarado R., Falcón M.D.C.P.T. (2021). Self-Tuning Neural Network PID with Dynamic Response Control. IEEE Access.

[26] Bari S., Hamdani S.S.Z., Khan H.U., Rehman M.u., Khan H. Artificial Neural Network Based Self-Tuned PID Controller for Flight Control of Quadcopter. Proceedings of the International Conference on Engineering and Emerging Technologies (ICEET).

[27] Gómez-Avila J., López-Franco C., Alanis A.Y., Arana-Daniel N. Control of Quadrotor using a Neural Network based PID. Proceedings of the IEEE Latin American Conference on Computational Intelligence (LA-CCI).

[28] Esim E., Yıldırım Ş. (2017). Drilling performance analysis of drill column machine using proposed neural networks. Neural Comput. Appl..

[29] Eski I., Erkaya S., Savas S., Yildirim S. (2011). Fault detection on robot manipulators using artificial neural networks. Robot. Comput.-Integr. Manuf..

[30] Jesus O.D., Hagan M.T. (2007). Backpropagation Algorithms for a Broad Class of Dynamic Networks. IEEE Trans. Neural Netw..

[31] Shohda A.M.A., Ali M.A.M., Ren G., Kim J.-G., Mohamed M.A.-E.-H. (2022). Application of Cascade Forward Backpropagation Neural Networks for Selecting Mining Methods. Sustainability.

[32] Tengeleng S., Armand N. (2014). Performance of Using Cascade Forward Back Propagation Neural Networks for Estimating Rain Parameters with Rain Drop Size Distribution. Atmosphere.

[33] Loy J. (2019). Neural Network Projects with Python: The Ultimate Guide to Using Python to Explore the True Power of Neural Networks through Six Projects.

[34] Sohrabi P., Jodeiri Shokri B., Dehghani H. (2023). Predicting coal price using time series methods and combination of radial basis function (RBF) neural network with time series. Miner. Econ..

[35] Deng Y., Zhou X., Shen J., Xiao G., Hong H., Lin H., Wu F., Liao B.-Q. (2021). New methods based on back propagation (BP) and radial basis function (RBF) artificial neural networks (ANNs) for predicting the occurrence of haloketones in tap water. Sci. Total Environ..

[36] Zhou S., Yang C., Su Z., Yu P., Jiao J. (2023). An Aeromagnetic Compensation Algorithm Based on Radial Basis Function Artificial Neural Network. Appl. Sci..

[37] He H., Yan Y., Chen T., Cheng P. (2019). Tree Height Estimation of Forest Plantation in Mountainous Terrain from Bare-Earth Points Using a DoG-Coupled Radial Basis Function Neural Network. Remote Sens..

